# Biosynthesis of uniformly carbon isotope-labeled docosahexaenoic acid in *Crypthecodinium cohnii*

**DOI:** 10.1186/s13568-020-00981-0

**Published:** 2020-03-11

**Authors:** Pingping Song, Alexander Kuryatov, Paul H. Axelsen

**Affiliations:** 1 School of Biological Engineering, Guizhou Medical University, Guiyang, 550025 Guizhou China; 2 Department of Pharmacology, Biochemistry and Biophysics, University of Pennsylvania, Philadelphia, PA 19104 USA; 3 Department of Medicine, University of Pennsylvania, Philadelphia, PA 19104 USA

**Keywords:** *Crypthecodinium cohnii*, Docosahexaenoic acid, ω-3 polyunsaturated fatty acids, Carbon isotope labeling, Oxidative stress

## Abstract

Docosahexaenoic acid (DHA) enriched in brain can yield many important degradation products after the attack of hydroxyl radicals, which is known to serve as a nutraceutical and neuroprotective effects. Oxidative stress is a commonly observed feature of Alzheimer’s disease (AD). Therefore, uniformly radiolabeled DHA plays an important role in studying the oxidative fate of DHA in vivo and vitro. However, carbon isotope labeled DHA isn’t commercially available now. The heterotrophic microalgae *Crypthecodinium cohnii* (*C. cohnii*) has been identified as a prolific producer of DHA. In this study, the growth rate and DHA production in *C. cohnii* were optimized in a new defined media, and the biosynthesis of U-^13^C-DHA from U-^13^C-glucose and U-^14^C-DHA from U-^14^C-glucose were analyzed by HPLC–MS/MS. Approximately 40 nmoles of U-^13^C-DHA with higher isotopic purity of 96.8% was produced in a 300 μL batch, and ~ 0.23 μCi of U-^14^C-DHA with significant specific activity of 5–6 Ci/mol was produced in a 300 μL batch. It was found that *C. cohnii* had the optimal growth and DHA accumulation at 25 °C in this defined media (C/N = 10). An efficient protocol for the biosynthesis of U-^13^C-DHA and U-^14^C-DHA were set up firstly, which provides the basic support for the analysis of oxidative degradation products of DHA in AD.

## Introduction

Docosahexaenoic acid (DHA) as a predominant ω-3 polyunsaturated fatty acid (ω-3 PUFA) is known to play multi-functional roles in brain diseases (Sun et al. 2018; Harauma et al. 2017). DHA is rapidly accumulated in the brain during the gestation and early infancy, and the DHA availability from maternal stores can affect the DHA incorporation into neural tissues (Weiser et al. 2016). Some studies found that excessive DHA intake might modify the risk of brain diseases, hence appreciable amounts of DHA in the brain may serve as a nutraceutical and neuroprotective effects (Sun et al. 2018; Calon and Cole 2007).

Oxidative stress is a commonly observed feature of Alzheimer’s disease (AD) (Jiang et al. 2016; Namioka et al. 2017; Axelsen et al. 2011). PUFAs, such as arachidonic acid (ARA) and DHA, are abundant in brain and especially vulnerable to the attack of hydroxyl radical, which can induce the production of many degradation products (Corsinovi et al. 2011; Nowak 2013). ARA from membrane phospholipids can be released by phospholipase A_2_ in cytoplasm, meanwhile DHA is connected to action of the phospholipase A_2_. DHA can make enzymatic conversion by 15-lipoxygenase to form important lipid mediators including the resolvins and neuroprotectins (Strokin et al. 2004). DHA can also make non-enzymatic conversion by the oxygen free radicals (ROS), which would induce the production of 4-hydoxy-2-hexenal (HHE), an aldehyde derivative of ω-3 PUFA oxidation which can form adducts. It was found that 4-hydoxy-2-nonenal (HNE) as a product of ω-6 PUFA oxidation was elevated in AD, which could mediate the neurotoxicity of Aβ peptides and accelerate the fibril formation, but HHE has no this effect (Murray et al. 2005, 2007; Liu et al. 2008). Hence, the relative availability of ω-3/ω-6 substrates may play an important role in the induction of oxidative stress to Aβ fibril formation. To explore the relationship between oxidative stress and amyloid plaques, uniformly radiolabeled ARA and/or DHA need to be introduced into transgenic mouse models of AD via intracerebroventricular injection.

Stable isotope ^13^C-labeled PUFA standards have many advantages as research tools, because they may be distinguished from their naturally abundant counterparts by mass spectrometry and directly incorporated as internal standards into analytical procedures (Le et al. 2007). Currently, ^13^C-labeled PUFAs are expensive, available in limited supply, so marine heterotrophic microorganisms are being screened for PUFAs production. In our lab, U-^13^C- and U-^14^C-ARA from *Mortierella alpina* has been prepared with a high isotopic purity of 95% and used to the analysis of amyloid plaque-associated oxidative degradation production of ARA (Furman et al. 2016; Lee et al. 2017). Now uniform labeling with ^13^C and ^14^C would be used to quantify the oxidative degradation products of DHA in AD.

*Crypthecodinium cohnii* (*C. cohnii*) has been considered as a prolific producer of DHA. The heterotrophic microalgae is very amazing in that it can accumulate a high fraction of DHA with trivial amounts of other PUFAs in cell lipids, which makes the DHA isolation very attractive in pharmaceutical and nutraceutical applications (Udayan et al. 2017; Ziboh et al. 1970). Despite the importance of DHA, the pathways of fatty acid synthesis in *C. cohnii* still remain unclear. Some studies concluded that fatty acid synthetase (FAS) might provide the precursors for DHA biosynthesis in *C. cohnii* (Sonnenborn and Kunau 1982). De Swaaf conducted the ^13^C-NMR analysis for DHA biosynthesis by ^13^C-labeled externally supplied precursor (de Swaaf et al. 2003). They found that the biosynthesis of saturated fatty acids (SFA), the conversion of SFA to monounsaturated fatty acids and de novo synthesis of DHA may regulate the fatty acid production in *C. cohnii*.

The biosynthesis of partial ^13^C-labeled forms of DHA has been described previously, but for mass spectrometry it is required to have uniformly labeled forms of DHA with high isotopic purity as an internal standard. Even if d5-DHA that is commercially available is usually used for mass spectrometric quantitation of DHA, the deuterium atoms are liable and frequently lost during the chemical oxidation and enzymatic metabolism (Yasumoto et al. 2017). At present, there are no available U-^13^C- and ^14^C-DHA commercially. Glucose is the most commonly used substrate for lipid accumulation in microorganism and is available in uniformly isotopically labeled forms. Hence, *C. cohnii* was cultivated in a new synthetic media with a goal to the efficient biosynthetic production of U-^13^C- and U-^14^C-DHA using U-^13^C- and U-^14^C-glucose as a carbon source.

## Materials and methods

### Materials

U-^13^C-glucose was purchased from Cambridge Isotope Laboratories (Andover, MA, USA). U-^14^C-glucose (300 Ci/mol, 1 mCi/mL) was purchased from American Radiolabeled Chemicals (Saint Louis, MO, USA). DHA was purchased from Nu-Check Prep Inc. (Elysian, MN, USA). d5-DHA was purchased from Cayman Chemicals (Ann Arbor, Michigan). *Crypthecodinium cohnii* (ATCC 40750) was obtained from American Type Culture Collection (Manassas, VA, USA). All other chemicals were obtained from Sigma-Aldrich (St. Louis, MO, USA).

### Media and culture conditions

*Crypthecodinium cohnii* cells were grown in standing cultures (10 mL in 50 mL sterile tube) in complex media (4 g/L yeast extract, 12 g/L glucose, 35 g/L sea salt) at 26 °C in the dark. The inoculated OD_600_ were about 0.15. After 4–5 days, OD_600_ reached ~ 1.5, 1.5–2 mL of this culture were centrifuged at 500*g* for 1 min. The supernatants were discarded, the pellets were washed in ~ 2 mL of defined media (without glucose), centrifuged at 500*g* for 1 min, then resuspended in 10 mL of new chemically defined media (Table 1) with either 9 g/L ^13^C- or ^14^C/^12^C-mixed glucose. The final OD_600_ were adjusted to 0.15–0.2, and incubated at 26 °C for 6–8 days in the dark shaker (EDISON, NJ, USA) at 200 rpm.

**Table 1:** The ^14^C activity distribution of fatty acids in *C. cohnii*

	EHA	DHA	Palmitic acid	Oleic acid	Stearic acid
Activity (μCi)	3.34 ± 0.74	46.4 ± 2.64	15.43 ± 0.94	15.0 ± 0.27	5.77 ± 0.19
Conversion (%)	0.07	1.03	0.34	0.33	0.13
Elution time (min)	15.5	26	46	49	55.5
m/z (^13^C)	320	349	277	303	305

*EHA* eicosahexaenoic acid

The new defined media, originally developed by Tuttle and Loeblich (2019), contained per liter: 9 g glucose, 1 g K_2_HPO_4_, 10.6 g MgCl_2_·6H_2_O, 1.1 g CaCl_2_, 0.7 g KCl, 3.9 g Na_2_SO_4_, 0.1 g SrCl_2_·6H_2_O, 0.1 g KBr, 23.5 g NaCl, 0.2 g NaHCO_3_, 0.15 g disodium glycerophosphate, 1 g sodium glutamate, 5 mL metal mixture, 1 mL vitamin solution. The pH was adjusted to 6.4. The metal mixture in defined media contained per liter: 0.5 g FeCl_3_·6H_2_O, 10 g Na_2_EDTA, 10 g H_3_BO_3_, 0.01 g CoCl_2_·6H_2_O, 1.6 g MnCl_2_·4H_2_O, 0.1 g ZnCl_2_. The vitamin mixture in defined media contained per liter: 100 mg Thiamin, 5 mg Vitamin B_12_, 20 mg Aminobenzoate, 10 mg Ca pantothenate, 3 mg Biotin, 100 mg Riboflavin. All stock solutions were sterilized by filtration through 0.22 μm Milex syringe filters.

The *C. cohnii* cultures were incubated in the above culture conditions, and sampled everyday for analysis, 3 replicates per group were performed in this experiment.

### The growth rate and glucose consumption

The growth rate was determined by the OD_600_ with Cary 400 Bio UV–vis spectrophotometer (Agilent, Santa Clara, CA).

The glucose consumption was measured by the DNS method (Miller 1959). After the centrifugation of algal culture, the supernatant (25 µL) were taken and added to 275 µL water, then 300 µL DNS (containing 1 g/L 3,5-dinitrosalicylic acid, 0.1 g/L Na_2_SO_3_, 1 g/L NaOH) were added, incubated for 10 min at 90 °C. After the incubation, 600 µL of quencher (40 g/L sodium potassium tartrate) were added, and the final solution was cooled to room temperature. The OD_540_ were measured by the spectrophotometer, the glucose concentration was analyzed by the glucose standard curve (y = 0.9386x, R^2^ = 0.9931).

### Lipid extraction and saponification

#### Lipid extraction

300 µL of algal culture in 1.5 mL Eppendorf tube were centrifuged for 1 min at 2000*g*. The supernatant was removed, 640 µL of water were added to re-suspend the pellet. The suspension was subjected to three free-thaw cycles (liquid nitrogen alternating with boiling water), cooled down on the ice. 1.6 mL of methanol and 800 µL of dichloromethane were added and mixed, then sonicated 90 s on ice. 800 µL of dichloromethane and 640 µL of water were added to separate phases, which were centrifuged at 400*g* for 1 min. The lower phase was withdrawn and transferred to new 13 × 100 mm glass tubes, dried under argon.

#### Saponification

Samples were saponified in 85% methanol (1.5 mL) in water with 1 M NaOH (0.5 mL) at 80 °C for 1 h, and then cooled at room temperature. After that, they were acidified with 400 µL of 5 M HCl, then 1 mL of isooctane was added to extract for three times. Three upper phases were combined in glass tubes, and evaporated under argon. 100 µL of ethanol were added to dissolve the sample, and put in freezer (− 80 °C) after filling with the argon.

### HPLC separation and mass spectrometry analysis

#### DHA yield

5 µL samples were injected into a 1.0 × 50 mm Eclipse XD8-C18 3.5 µm column. The solvent A was 60% acetonitrile, 40% water and 0.1% formic acid. The solvent B was 100% acetonitrile and 0.1% formic acid. The mobile phase was pumped at 0.1 mL/min as the composition was changed linearly from 0 to 100% solvent B at 5–6.5 min, 100% solvent B at 6.5–10 min, returned to 0% at 10–12.5 min. The eluent on alkalinized post-column was 0.15 M ammonium hydroxide in methanol flowing at 50 µL/min, which was introduced into ABI 4000 Q1 Trap tandem mass spectrometer (Sciex, Toronto, Canada) via electrospray ionization in negative polarity. The declustering potential (DP) was − 100 V, the ionspray voltage (Is) was − 4200 V, the temperature of drying gas (TEM) was 300 °C, the collision energy (CE) was − 30 V and the collision gas (CAD) was 4psi for multiple reaction monitoring (MRM) mode. The m/z transitions in MRM mode were from 349.2 to 304.2 for ^13^C-DHA with the neutral loss of CO_2_, 332.2–288.2 for d5-DHA, 327.2–283.2 for ^12^C-DHA. d5-DHA as internal standard was added to lipid extracts to quantify the recovered U-^13^C-DHA. The efficiency (E) of U-^13^C-glucose conversion into U-^13^C-DHA in culture was calculated by using Eq. 1.

1 $${\text{E}} = \frac{{{\text{moles U-13}}_{\text{C}}{{\text{-DHA}}}*22}}{{{\text{moles U-13}}_{\text{C}}{{\text{-glucose}}}*6*{\text{P}}_{\text{iso}} }}$$

#### Isotopic purity

20 µL samples were injected into a 4.6 × 150 mm Eclipse XD8-C18 3.5 µm column. Ditto for the compositions of solvent A and B. The mobile phase was pumped at 0.5 mL/min as the composition was changed linearly from 40% solvent B at 0–10 min, 40–100% solvent B at 10–40 min, 100% solvent B at 40–50 min, finally returned to 40% at 50–60 min. The flowing rate of eluent on the post-column was 250 µL/min. The declustering potential (DP) was − 75 V, the ionspray voltage (Is) was − 4500 V, the temperature of drying gas (TEM) was 300 °C, the collision energy (CE) was − 10 V and the collision gas (CAD) was 7psi for Q1 or enhanced mass spectrometer (EMS) mode. The m/z transitions in EMS mode were from 324 to 352 for ^13^C-DHA. U-^13^C-DHA purified from *C. cohnii* was eluted as a single peak with the m/z of 327–349, depending on the number of ^13^C atoms in the molecule. The isotopic purity (P_iso_) of U-^13^C-DHA was calculated by Eq. 1 (Eq. 2), f_i_ is the integrated area of the peak at m/z = i.

2 $$P_{iso} = \frac{{\sum\nolimits_{327}^{349} {\left( {\frac{{{\text{i}} - 327}}{20}} \right){{\text{f}}}_{{\text{i}}} } }}{{\sum\nolimits_{327}^{349} {{{\text{f}}}_{{\text{i}}} } }}$$

#### DHA purification

Crude fatty acids of 120 µL from *C. cohnii* was injected into 150 µL loop and run using the 4.6 × 150 mm Eclipse XD8-C18 3.5 µm column by the HPLC. Ditto for the compositions of solvent A and B. The mobile phase was pumped at 0.5 mL/min. The gradient program were 40% solvent B at 0–2 min, 40–50% solvent B at 2–10 min, 50–100% solvent B at 10–45 min, 100% solvent B at 45–54 min, finally returned to 40% at 54–60 min. All fractions were collected into 1.5 mL Eppendorf tubes by the Automatic Fraction Collector (BECKMAN, SC 100), one fraction per 0.5 min. 5 µL solution from every fraction and 495 µL ethanol were mixed, and then run by the HPLC–MS. The fractions containing DHA were dried by the argon and put in freezer (− 80 °C) after filling with the argon.

### The statistical analysis

Based on obtained data, the mean and standard deviation of three parallel samples per group were calculated, and one-way analysis of variance was conducted by using the SPSS 19.0 software. p < 0.05 indicates that the two groups have the difference, p < 0.01 indicates the two groups have significant difference.

## Results

### The U-^13^C-DHA production of *C. cohnii* in defined media

#### The growth rate and U-^13^C-DHA production

**Fig. 1:**
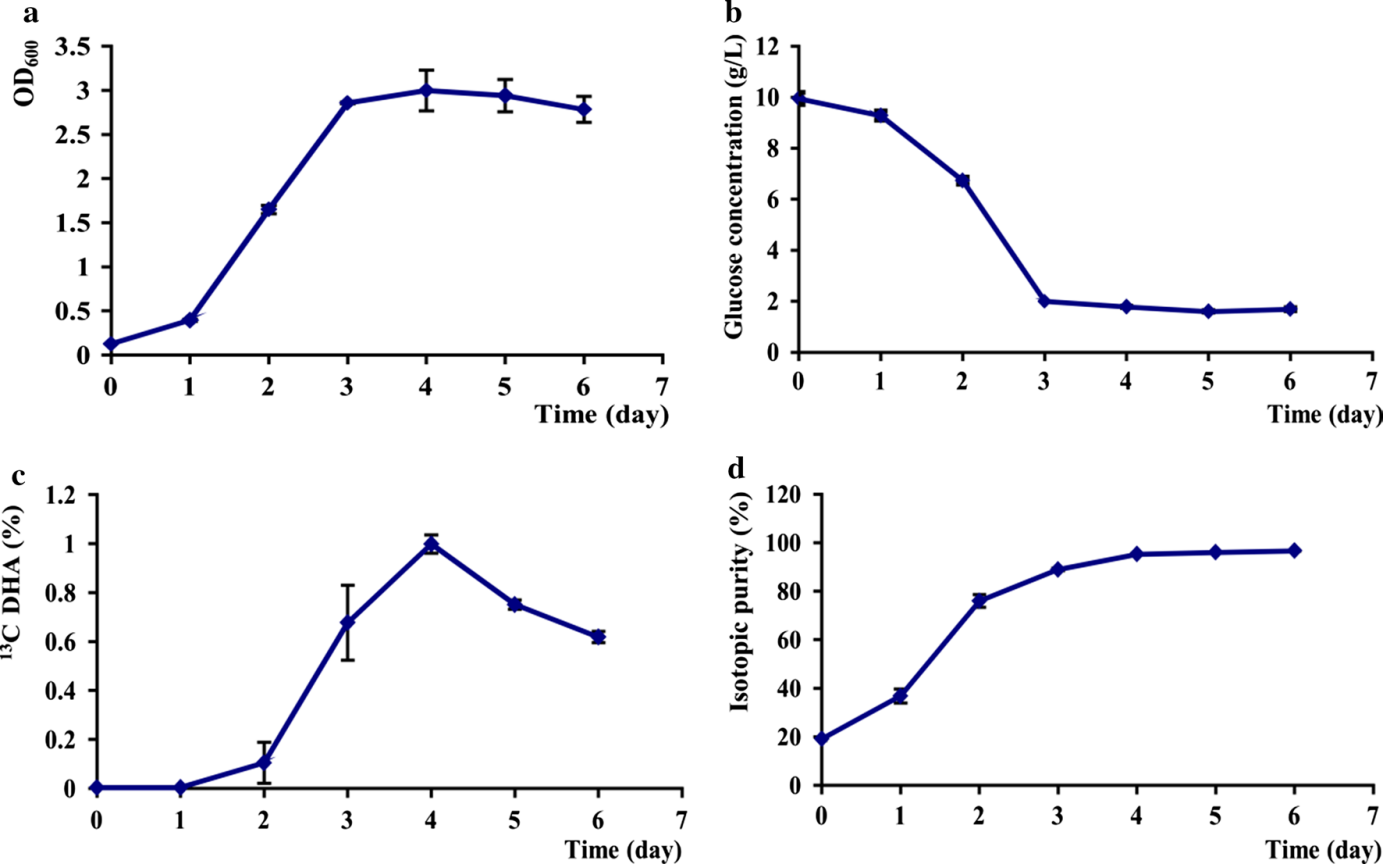
The growth rate and U-^13^C-DHA production in *C. cohnii*. **a** OD_600_; **b** glucose consumption; **c** U-^13^C-DHA yield; **d** isotopic purity

After the inoculation, day 1 was latent phase, the algal cells nearly didn’t grow. 1–3 days were logarithmic phase, the growth rate was accelerated significantly. After day 3, *C. cohnii* entered the stationary phase, and reached a maximum OD_600_ (~ 3) on day 4, declined somewhat on day 6 (Fig. 1a, p < 0.05). Meanwhile, the glucose consumption in *C. cohnii* was also very rapid over days 1–3, and then stopped in 2 g/L on days 4–6 (Fig. 1b).

During the culture period, U-^13^C-DHA yield in *C. cohnii* were nearly zero on day 1, slightly increased to 0.1% on day 2. Afterwards, U-^13^C-DHA accumulation in algal cells was accelerated and reached a maximum of ~ 1% on day 4, but declined significantly after day 5 (Eq. 1, Fig. 1c, p < 0.01). The original isotopic purity of U-^13^C-DHA in *C. cohnii* was very low. On days 1–4, U-^13^C-DHA isotopic purity increased sharply from 39 to 96.8%, then kept in stable level on days 5–6 (Eq. 2, Fig. 1d).

#### The mass spectrums of U-^13^C-DHA

**Fig. 2:**
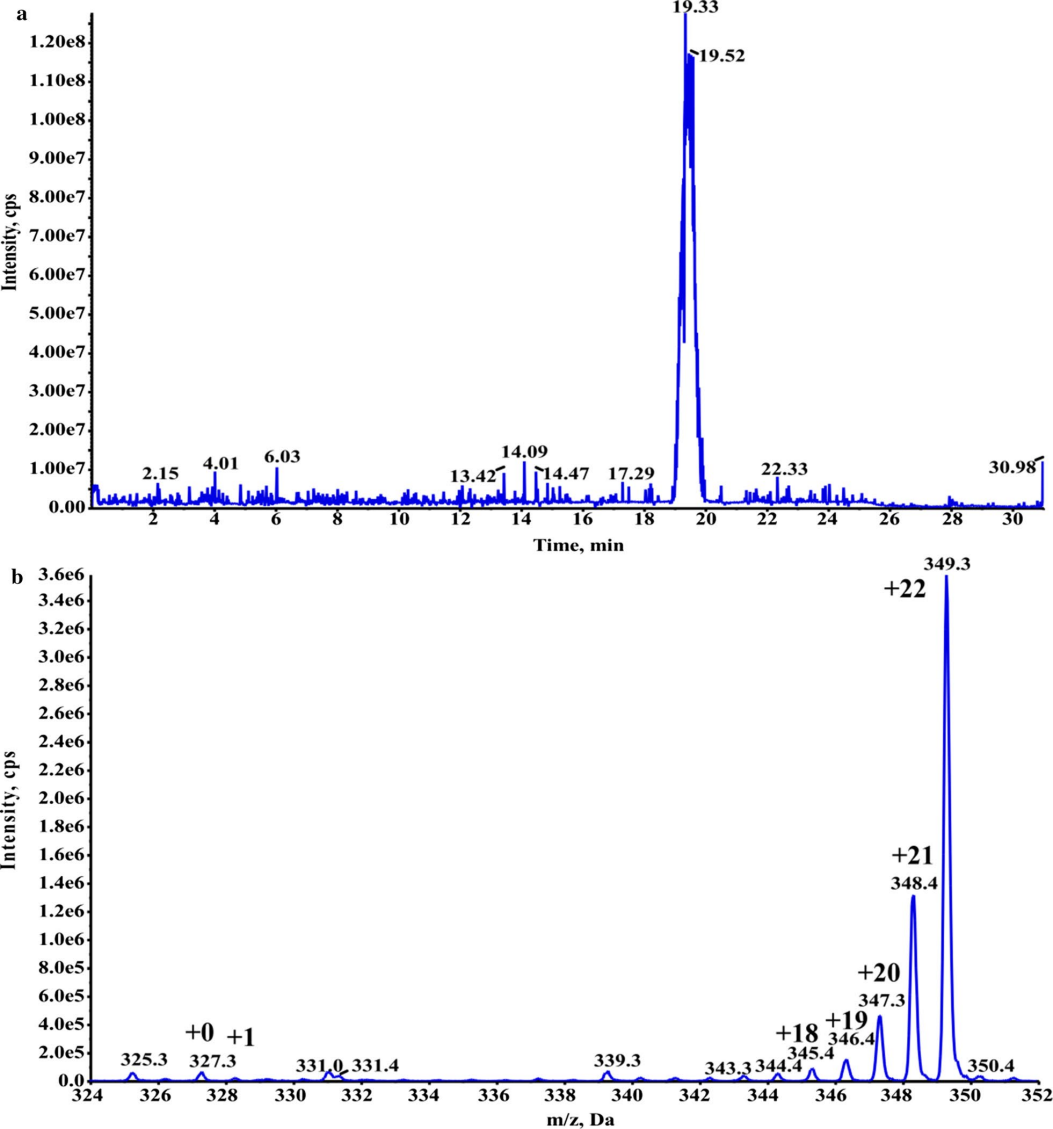
The mass spectrums of purified U-^13^C-DHA from *C. cohnii* in the EMS mode. **a** Total ion current of the lipid extracts collected from HPLC fractions between 24–27 min for m/z = 324–352; **b** unlabeled and labeled ^13^C-DHA

The lipid extracts collected from HPLC fractions between 24 and 27 min were analyzed. The total ion current of purified U-^13^C-DHA was got at m/z = 324–352 in EMS mode. The single peak appeared at 19–20 min with the highest CPS intensity of 1.25e^8^. The m/z (327) in unlabeled DHA was consistent with the natural isotopic abundances of carbon, and the CPS intensity was very low. The m/z (345–349) in U-^13^C-DHA were corresponding to isotope labeling carbons of 18–22 respectively. + 0 was unlabeled DHA, + 1 was DHA with one ^13^C atom, + 18 to + 22 were DHA with 18–22 ^13^C atoms (Fig. 2a, b).

### The U-^14^C-DHA production of *C. cohnii* in defined media

#### The grow rate and U-^14^C-DHA production

**Fig. 3:**
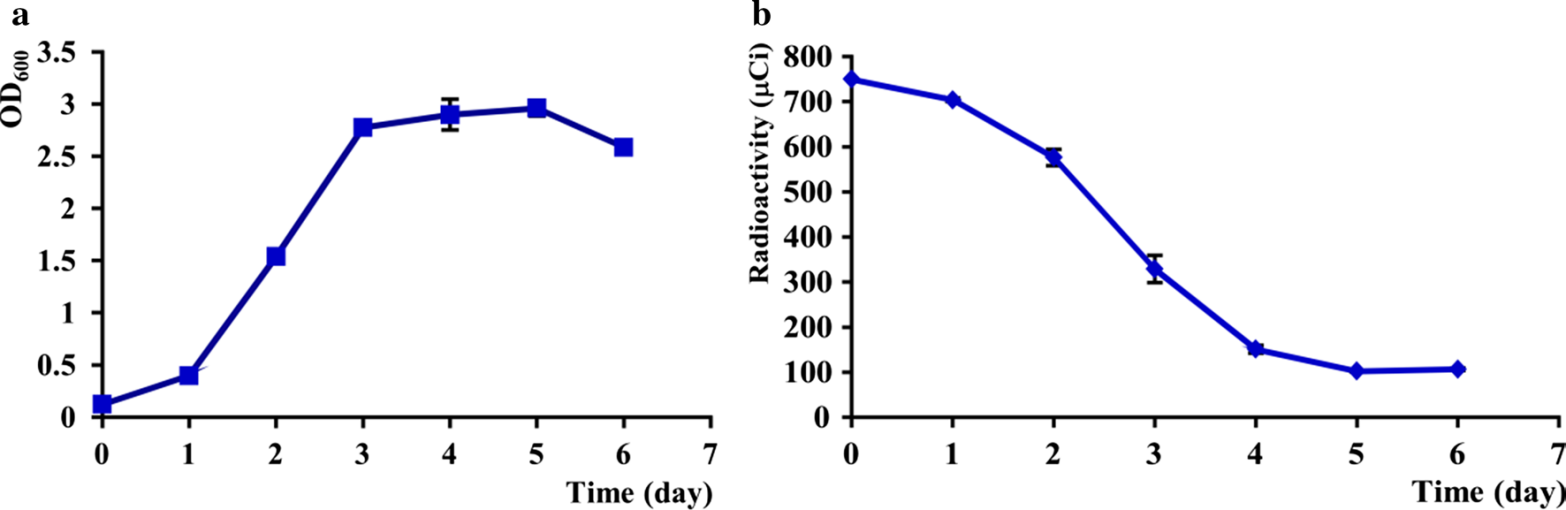
The growth rate and U-^14^C-DHA production in *C. cohnii*. **a** OD_600_; **b** glucose consumption. 300 μL/batch

During the culture period, algal growth state in U-^14^C-labeling defined mediawas very similar with the growth in the U-^13^C-labeling defined media. After day 3, *C. cohnii* also entered the plateau, kept higher OD_600_ (~ 3) on days 4–5, and declined on day 6 (Fig. 3a, p < 0.05). Meanwhile, glucose in culture was also consumed rapidly on days 1–4 and stopped in 100 μCi on days 5–6 (Fig. 3b). The radioactivity was used to analyze the algal glucose consumption.

U-^14^C-DHA from *C. cohnii* was quantified by scintillation counting. To estimate the mass of U-^14^C-DHA, U-^13^C-DHA was produced under same conditions and quantified by mass spectrometry. The EMS scan revealed that the isotopic purity of DHA containing 18–22 ^13^C atoms (m/z = 345–349) reached a maximum of 96.8% (Eq. 2, Fig. 2). ~ 40 nmoles of U-^13^C-DHA was recovered for a conversion efficiency of 1% (Eq. 1). The yield suggested that the specific activity of U-^14^C-DHA was approximately 5-6 Ci/mol, which was calculated by the equation (A*P_iso_)/moles U-^13^C-DHA. A is the total activity of ^14^C-DHA.

#### The activity distribution of ^14^C-labeling fatty acids

**Fig. 4:**
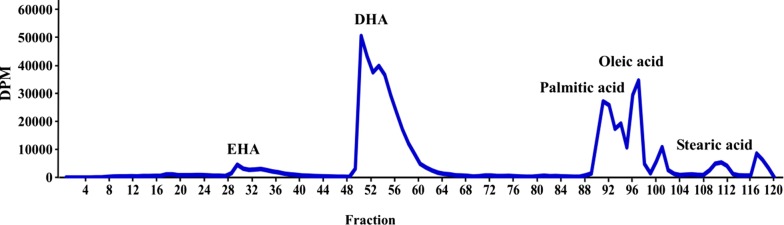
The activity map for the ^14^C-labeling fatty acids in *C. cohnii*. Five fatty acids in different elution times were marked out. Totally 120 fractions were collected, 0.5 min/per fraction. Five parallel samples were analyzed in same conditions

The activities of ^14^C-labeling fatty acids in *C. cohnii* were analyzed by mass spectrometry and scintillation counting. Totally 120 fractions from algal lipid extracts were collected. The U-^14^C-DHA activity was the highest (46.46 μCi) at 26 min. In addition to DHA, four other fatty acids in different elution times were also identified, including EHA (15.5 min), palmitic acid (46 min), oleic acid (49 min) and stearic acid (55.5 min) (Fig. 4, Table 1).

## Discussion

In previous experiments, it was found that *C. cohnii* had the optimal growth and DHA accumulation in 25 °C, 0.2 inoculated density, 10:1 C/N, and 5:1 air/culture volume ratio in a new defined media. Hence, in this experiment, *C. cohnii* was cultivated in the same conditions, but carbon isotope labeling defined media were used for the biosynthesis of U-^13^C-DHA and U-^14^C-DHA.

During the culture period, the growth rate in *C. cohnii* reached a maximum OD_600_ (~ 3) on day 4. Meanwhile, glucose consumption stopped in 2 g/L on days 5–6, and the U-^13^C-DHA yield also reached a maximum of 1% on day 4 (Fig. 1). The U-^13^C-DHA yield of algal cells was measured by HPLC and mass spectrometry in MRM mode, and d5-DHA as a calibrated internal standard was added to lipid extracts. The isotopic purity of U-^13^C-DHA is very important in mass spectrometric quantitation, which relies on complete isotope substitution in both parent ion and collision-induced fragments (Hineman et al. 1993). In current experiment, the isotopic purity was maximized by omitting various ^12^C sources in the media, such as yeast extract, and substituting the inorganic nitrogen source. The isotopic purity of commercially-prepared U-^13^C-glucose was 99%, but the final isotopic purity of recovered DHA in *C. cohnii* reached a maximum of 96.8% on day 4 (Eq. 2, Fig. 1d). As *C. cohnii* was nonphotosynthetic and obligate heterotrophs, the most likely contribution of unlabeled carbon comes from the glutamate used as the sole nitrogen source in the ^13^C media. For example, the production of the TCA cycle intermediate α-ketoglutarate following transamination in amino acid synthesis (Le et al. 2007). The total ion current of purified U-^13^C-DHA was got at m/z = 324–352 in EMS mode. The m/z (327) in unlabeled DHA was consistent with the natural isotopic abundances of carbon. The m/z (345–349) in U-^13^C-DHA were corresponding to isotope labeling carbons of 18–22 (Fig. 2). The production of U-^13^C-DHA from U-^13^C-glucose in *Hyalochlorella marina* has been reported, but the yield was lower and the isotopic purity was only ~ 90% (Le et al. 2007; Chouinard-Watkins et al. 2013).

Algal growth state in defined media containing ^14^C-glucose was very similar with the U-^13^C-defined media. U-^14^C-glucose was supplied with a maximal specific activity of 300 Ci/mol (1 mCi/mL, 5 mL). 0.75 mCi of U-^14^C-glucose was mixed with 0.5 mmoles of ^12^C-glucose in 10 mL culture to make the final glucose concentration of 9 g/L. Totally U-^14^C-glucose was diluted 200 times with ^12^C-glucose. In this media, *C. cohnii* in the plateau kept higher OD_600_ (~ 3) on days 4–5. Meanwhile, the U-^14^C-glucose radioactivity in culture declined sharply and stopped in 100 μCi on days 5–6 (Fig. 3), which was quantified by liquid scintillation counting. To estimate the isotopic purity and specific activity of the radiolabeled material, U-^13^C-DHA was produced under the same conditions and quantified by MRM and EMS mass spectrometry after adding the d5-DHA. The EMS scan revealed that DHA contained 18–22 ^13^C atoms (m/z = 345–349) for a maximal isotopic purity of 96.8% (Eq. 2, Fig. 2). From three separate cultures (300 μL/batch), each contained 14.5 μmoles of U-^13^C-glucose, so averagely 40 nmoles of U-^13^C-DHA was recovered for a conversion efficiency of 1% (Eq. 1). The yield suggested that the specific activity of U-^14^C-DHA may have been as high as ~ 5–6 Ci/mol, which was close to the theoretical maximum of 5.5 Ci/mol (0.25 Ci/mol/carbon). Previous research found that ^14^C-labeled oleic acid was detected in *C. cohnii*, but not DHA (Beach et al. 1974). In contrast, other researchers only detected a small amount of ^14^C-labeled DHA (Henderson and Mackinlay 1991). In addition to DHA, four other fatty acids in *C. cohnii* were also identified by mass spectrometry, and their ^14^C activities were measured by the liquid scintillation counting (Fig. 4, Table 1). The total activity of four fatty acids were ~ 39.6 μCi, and the overall conversion efficiency for U-^14^C-glucose into fatty acids were about 1.9%, of which 54.2% was DHA.

At present, the effectiveness of U-^13^C-DHA has been tested on healthy older persons (Plourde et al. 2014). The authors were able successfully to trace significant modifications of kinetics of ^13^C-DHA when the participants were orally ^13^C-DHA supplement. In our experiments, U-^13^C-DHA and U-^14^C-DHA in *C. cohnii* were efficiently produced from isotope-labeling glucose on a laboratory scale. Purified U-^13^C-DHA and U-^14^C-DHA with higher purity play an important role in exploring the relationship between oxidative stress and amyloid plaques, which will be used not only for analysis of DHA oxidative fate in brain, but also for intracerebroventricular injection to transgenic mouse models of AD.

## Data Availability

The data and materials in the study are shared and available.
